# Developments in Biological Mechanisms and Treatments for Negative Symptoms and Cognitive Dysfunction of Schizophrenia

**DOI:** 10.1007/s12264-021-00740-6

**Published:** 2021-07-05

**Authors:** Qiongqiong Wu, Xiaoyi Wang, Ying Wang, Yu-Jun Long, Jing-Ping Zhao, Ren-Rong Wu

**Affiliations:** 1 National Clinical Research Center for Mental Disorders, Department of Psychiatry, The Second Xiangya Hospital of Central South University, Changsha, 410011 China; 2 Shanghai Institutes for Biological Sciences, Chinese Academy of Sciences, Shanghai, 200031 China

**Keywords:** Schizophrenia, Genetics, Epigenetics, Negative symptoms, Cognitive dysfunction, Physical therapy

## Abstract

The causal mechanisms and treatment for the negative symptoms and cognitive dysfunction in schizophrenia are the main issues attracting the attention of psychiatrists over the last decade. The first part of this review summarizes the pathogenesis of schizophrenia, especially the negative symptoms and cognitive dysfunction from the perspectives of genetics and epigenetics. The second part describes the novel medications and several advanced physical therapies (e.g., transcranial magnetic stimulation and transcranial direct current stimulation) for the negative symptoms and cognitive dysfunction that will optimize the therapeutic strategy for patients with schizophrenia in future.

## Introduction

Schizophrenia is a group of major mental spectrum disorders of unknown etiology, with a lifetime prevalence of approximately 1% in the world population [1]. The main clinical manifestations include positive symptoms (delusions, hallucinations, and disorganized speech), negative symptoms (diminished emotional expression or avolition), and cognitive dysfunction (deficits in attention, working memory, and executive function). In the late stages, the long-lasting negative symptoms and cognitive dysfunction deprive patients of independent lives and impose great economic burdens on families and society [2].

Negative symptoms are prevalent in patients with schizophrenia as nearly 60% of outpatients present them throughout the disease process [3–7]. Furthermore, patients with prominent negative symptoms (i.e., with greater severity than the co-occurring positive symptoms) account for 40% of schizophrenia outpatients, of whom 19% do not even present prominent positive symptoms [8]. Besides, negative symptoms tend to last for a long time and worsen with time. This is because, firstly, 73% of patients with negative symptoms present with asociality and trait anhedonia in their early childhood/adolescence, before the onset of schizophrenia [9–12]; and secondly, a greater percentage of patients exhibit more stable deficits (from 67% to 80%) as schizophrenia progresses [13]. In general, negative symptoms are linked with poor functional outcomes and a heavy economic burden of patients with schizophrenia [14].

Patients with schizophrenia who present cognitive impairments account for 98.1% [15], and on average their cognitive performance is 1–2 standard deviations below controls [16, 17]. The cognitive dysfunction occurs nearly one decade earlier than the first onset of psychosis and worsens with progression of the disease [18]. Thus, the early cognitive decline is acknowledged as a hallmark feature of schizophrenia, and schizophrenia may be a cognitive illness [19, 20]. Furthermore, cognitive dysfunction is treated as one of the strongest predictors of functional recovery from schizophrenia. Because it had direct effects on patients’ quality of life, the efficacy of psychosocial rehabilitation programs, and employment retention [21–23].

## The General Mechanisms Underlying Negative Symptoms and Cognitive Dysfunction

The conceptualization of negative symptoms has developed a lot to understand its mechanisms [24]. There are generally two dimensions in negative symptoms including five domains: (1) the reduced expression dimension, including blunted affect and alogia; (2) the reduced motivation dimension (also named apathy dimension), including asociality, avolition, and anhedonia [14]. However, the neurobiology of negative symptoms remains mysterious. The general mechanisms are associated with deficits in dopamine transmission in mesocortical pathways and deficits in other neurotransmitter systems such as serotonergic, noradrenergic, and glutamatergic transmission [14]. Of note, dopamine D3 receptors play a critical role in regulating negative symptoms. The D3 receptors, localized in the prefrontal cortex and the nucleus accumbens, which control reward and motivation [25], regulate glutamatergic circuits connecting the prefrontal cortex to subcortical areas [26, 27].

**Fig. 1:**
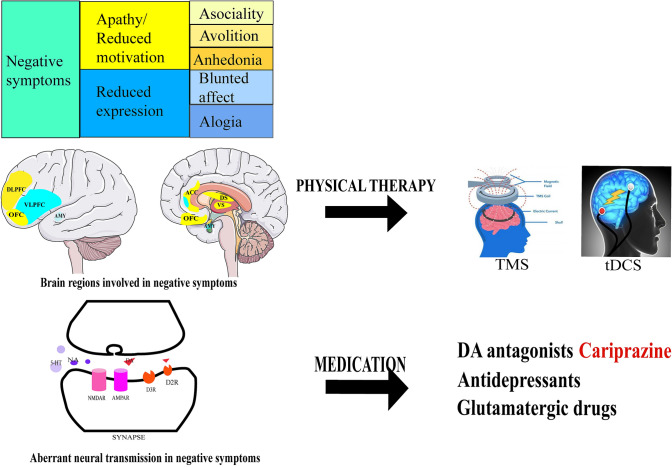
The pathophysiology and treatment of negative symptoms. The negative symptoms have two dimensions, apathy/reduced motivation and reduced expression. The apathy dimension (yellow) contains the asociality, avolition, and anhedonia domains, and the reduced expression dimension (blue) contains the blunted affect and alogia domains. The brain regions involved in the apathy dimension (yellow) include the anterior cingulate cortex (ACC), dorsal striatum (DS), dorsolateral prefrontal cortex (DLPFC), orbito-frontal cortex (OFC), and ventral striatum (VS). The brain regions involved in the reduced expression dimension (blue) include the ventrolateral prefrontal cortex (VLPFC) and amygdala (AMY). Physical therapy such as transcranial magnetic stimulation (TMS) and transcranial direct current stimulation (tDCS) may target these brain regions and improve negative symptoms. The abnormal synaptic transmission underlying the negative symptoms include dopaminergic, glutamatergic, and serotonergic transmission. The corresponding medications include dopamine antagonists, antidepressants, and glutamatergic drugs.

A meta-analysis of functional brain imaging studies reported that the ventral striatum hypoactivation during reward anticipation plays a central role in the negative symptoms of schizophrenia [28]. Different neural networks are involved in the different dimensions of negative symptoms (Fig. 1) [29]. For example, prefrontal-striatal networks (the ventral and dorsal striatum, ventromedial prefrontal cortex/orbito-frontal cortex, dorsolateral prefrontal cortex (DLPFC), and anterior cingulate cortex) play critical roles in the apathy dimension [29]. Other brain regions such as the rostral anterior cingulate cortex, amygdala, and basal ganglia are involved in the pathophysiology of the reduced expression dimension [29].

**Fig. 2:**
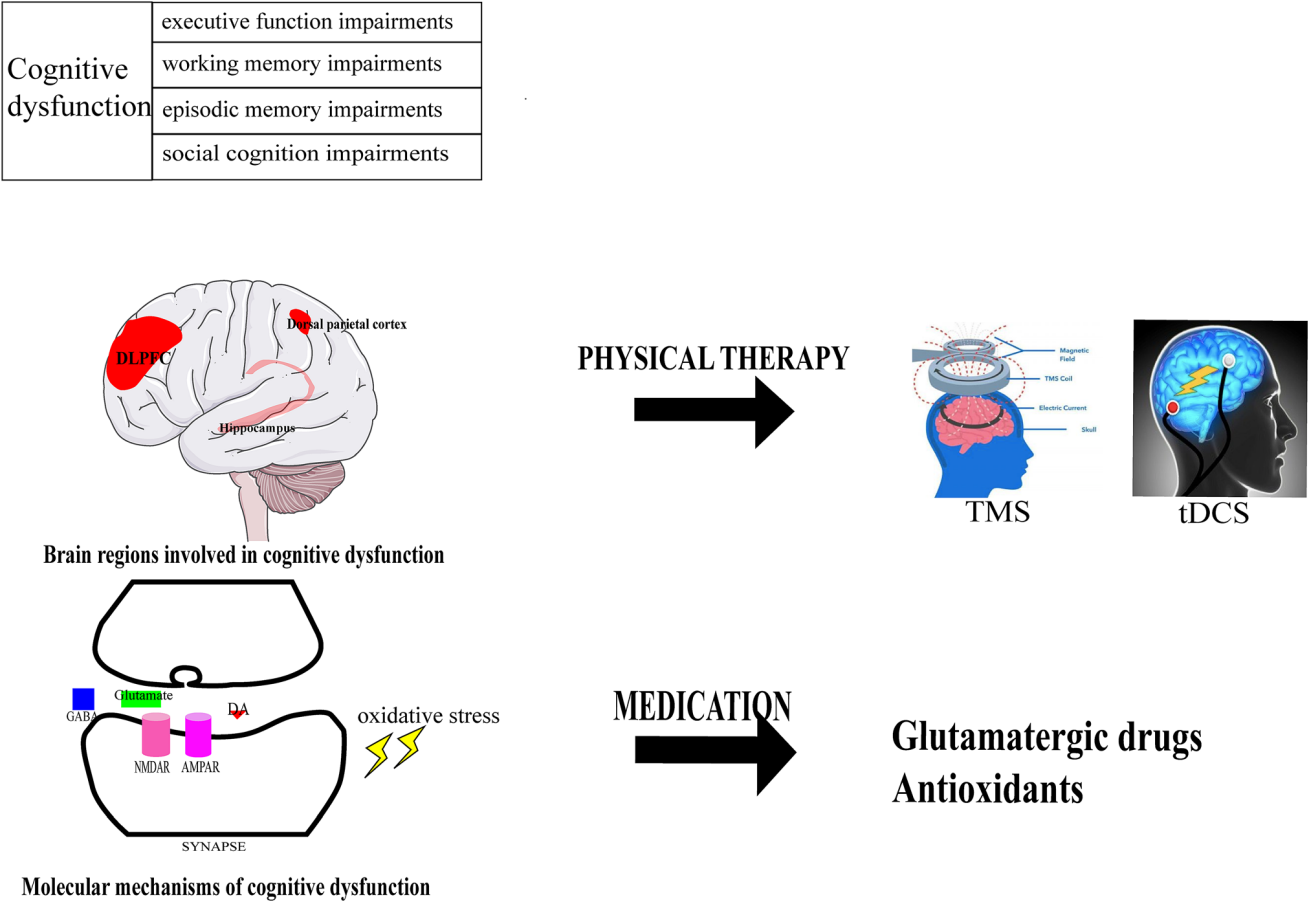
The pathophysiology and treatments of cognitive dysfunction. The cognitive dysfunction in schizophrenia is characterized by impairments of executive function, working memory, episodic memory, and social cognition. The brain regions involved in cognitive dysfunction include the hippocampus, dorsolateral prefrontal cortex (DLPFC), and dorsal parietal cortex. Physical therapy such as TMS and tDCS may target these brain regions and improve cognitive dysfunction in schizophrenia. The molecular mechanisms involved in cognitive dysfunction include abnormal glutamatergic transmission and oxidative stress. The corresponding medications include glutamatergic drugs and antioxidants.

Cognition includes eight domains: (1) attention/vigilance, (2) cognitive control/executive function, (3) reasoning/problem-solving, (4) social cognition, (5) speed of processing, (6) verbal learning, (7) visual learning, and (8) working memory [30, 31]. Among these, impairments in learning and memory, working memory, attention, problem solving, processing speed, and social cognition are prominent in schizophrenia [32, 33].The brain regions involved in cognition are the hippocampus, basal ganglia, DLPFC, and dorsal parietal cortex [34] (Fig. 2). Abnormalities in the cortico-cerebellar-striatal-thalamic loop, and task-positive and task-negative cortical networks have been identified in the functional connectivity analysis of resting-state functional magnetic resonance imaging in schizophrenia [35]. Of note, the impairments in context processing, working memory, and episodic memory are associated with impaired function of the DLPFC in schizophrenia [36]. Besides, episodic memory deficits have been highlighted in the cognitive dysfunction of schizophrenia as they are controlled by the DLPFC in the frontal and medial temporal lobe network [36]. Presynaptic and postsynaptic signaling has different effects on cognition, as the synthesis of presynaptic components is associated with memory, while postsynaptic signaling is associated with working memory [37]. The dysregulation of transmitters is involved in the cognitive dysfunction of schizophrenia as well. For example, the aberrant glutamatergic pathway might affect executive functioning, especially in cognitive flexibility and learning potential, [37] and an aberrant central muscarinic system (especially M1 and M4 receptors) has been described in regions linked to cognitive function [38].

Some associations between negative symptoms and cognitive symptoms have been found in schizophrenia. Conceptually, negative symptoms have some overlaps with cognitive dysfunction in schizophrenia [14]. For example, executive function in cognition affects goal-directed behavior, which contributes to avolition. The diminished verbal fluency and social cognition have some overlaps with alogia and asociality [24]. In addition to the overlapping concepts, cognitive impairment and negative symptoms have some mechanisms in common. For example, they may share the mechanism of aberrant interhemispheric connections (especially in the pallidum), as revealed in the fMRI of resting-state functional connectivity in first-episode schizophrenia patients [39]. Among molecular mechanisms, some glutamatergic variants influence both negative symptoms and cognitive dysfunction in the prediction analysis of groups at clinical high risk [40]. Besides, neuroinflammation and oxidative stress also play important roles in the pathogenesis of these two symptoms. Aberrant inflammatory status has been identified in patients with schizophrenia, and is associated with the severity of negative symptoms and cognitive dysfunction [41, 42]. Increased inflammatory processes such as microglial activation and elevated cytokine levels might have disruptive effects on neurogenesis and synaptic signaling [43]. In terms of oxidative stress, parvalbumin interneurons are vulnerable to oxidative stress, and the disruption of cortical and hippocampal parvalbumin interneurons may play key roles in the pathology of negative symptoms and cognitive dysfunction [44]. Plasma markers of oxidative stress (e.g., glutathione/GSH) interact with brain-derived neurotrophic factor and then impair cognition by affecting executive dysfunction in schizophrenia [45].

### Genetic Mechanisms of Negative Symptoms and Cognitive Dysfunction

As the neurodevelopmental model of schizophrenia has been proposed and refined with evidence from genetics and epigenetics, the mechanisms underlying negative symptoms and cognitive dysfunction have also been revealed by novel evidence from these fields. Multiple kinds of common variation [e.g., single nucleotide polymorphisms (SNPs)] and rare variation [e.g., copy number variants (CNVs) [46], loss-of-function variants [47, 48], and ultra-rare variants [49]] have been identified in schizophrenia. In general, SNPs are the primary common genetic variants with small effects while rare variants (including CNVs) generally have large effects [49]. For SNPs, common variant associations with the onset and treatment of schizophrenia have been identified in genome-wide association studies (GWASs) [50, 51]. In terms of rare variation, several CNV loci associated with the risk of schizophrenia and the medication response have been identified in GWASs [52–54].

The etiologies of negative symptoms have been associated with differential “modifier loci” in GWASs of schizophrenia [55]. In a meta-analysis of GWASs of Irish cases [55], SNPs in several genes (related to neuron projection and postsynaptic membrane) and several pathways (related to axon guidance, Netrin-1 signaling, and long-term depression) were demonstrated in negative symptoms [55]. Five SNPs (three SNPs in the *BCL9* gene and two in *C9orf5*) have robust associations with negative symptoms [56]. Besides, variation in other genes such as *DRD2*, which encodes the dopamine receptor DR2 [57], a *FOXO6* risk allele [58], and the catechol-O-methyltransferase (*COMT*) gene [59] are associated with the severity of negative symptom in patients with schizophrenia.

In the genetic mechanisms of cognitive dysfunction, several common and rare variations play important parts as well. SNPs of the psychosis susceptibility gene *ZNF804A* pathway are associated with IQ, memory, and social cognition in schizophrenia [60, 61]. Some CNVs such as the chromosome 15q11.2 deletion between breakpoints 1 and 2 diminish several cognitive traits and subsequently cause dyslexia and dyscalculia [62]. In addition to reports of several SNPs and CNVs in cognitive dysfunction, researchers have found that cognition and schizophrenia share some genetic bases [63], and several genetic loci jointly affect the risk of schizophrenia and cognitive traits (verbal-numerical reasoning, reaction time, and general cognitive function) [64]. Besides, there are negative correlations between genomic risk/polygenetic risk scores [65] for schizophrenia and cognitive traits such as general cognitive function [66] and IQ [67]. But this correlation is complicated and controversial as another two studies denied any association between polygenetic risk scores for schizophrenia and cognitive phenotypes [68, 69].

### Epigenetic Mechanisms of Negative Symptoms and Cognitive Dysfunction

Epigenetics refers to heritable phenotype changes without alterations in the DNA sequence. Much genetic risk cannot be explained at the DNA level and may be associated with epigenetic mechanisms in schizophrenia as well as its negative symptoms and cognitive dysfunction [1, 70].

First, schizophrenia is associated with DNA methylation changes at sites such as CpGs. Different brain regions may have different DNA methylation signatures in schizophrenia [71, 72], and a 4-fold enrichment is exhibited in schizophrenia risk loci among DNA methylation quantitative trait loci in the developing brain [73, 74]. In the negative symptoms, changes in DNA methylation in the oxytocin receptor gene are associated with the functional connectivity of the striatal-amygdala network underlying anhedonia-asociality in resting-state functional magnetic resonance imaging [75]. Patients with primary and enduring negative symptoms have lower DNA methylation in exons 4 and 5 of the matrix metalloproteinase 9 gene than other patients [76]. In cognitive dysfunction, CpG site methylation plays a key role in central nervous system differentiation as well as synaptic plasticity, learning, and memory [77]. Besides, hypomethylation of the *GRIN2B* promoter associated with cognitive dysfunction has been identified in early-onset schizophrenia *versus* controls [78]. In the DLPFC region associated with cognition, two expression and two methylation modules have been identified in schizophrenia [79]. Second, the spatial organization of genome architecture is critical in neurodevelopment as well as cognition in schizophrenia [80–82]. Schahram *et al*. found that the spatial organization of the genome architecture affects genes associated with the susceptibility to schizophrenia during the differentiation of neural progenitor cells, and these genes might affect risk variants at the transcriptome and proteome levels [83]. Third, some non-coding RNAs (e.g., long non-coding RNAs) are associated with the negative symptoms and cognitive performance [84] in schizophrenia [85].

The interactions among environmental risk factors, genetic mechanisms, and epigenetic mechanisms are: common genetic variations increase individuals’ susceptibility to environmental risk factors [86]; and environmental risk factors in early development alter the epigenetic regulation in schizophrenia [87–90]. The epigenetic mechanisms of negative symptoms and cognitive dysfunction are affected by environmental risk factors in schizophrenia. Patients with the *COMT Val(158) Met* polymorphism might have more severe negative symptoms if they suffer from emotional neglect [91]. Early-life diet can regulate learning and memory processes associated with schizophrenia *via* epigenetic mechanisms [92].

## Treatment of Negative Symptoms and Cognitive Dysfunction

Nowadays, antipsychotics are still the main treatment for schizophrenia [93]. The application of first-generation antipsychotics (FGAs/typical antipsychotics) is limited by their adverse effects on the motor system (e.g., adverse extrapyramidal effects) and the cardiovascular system. With the advent of second-generation antipsychotics (SGAs/atypical antipsychotics), the incidence of the side-effects noted above is lower but the incidence of severe metabolic disorders is higher [94]. Antipsychotics have a robust effectiveness in the treatment of acute schizophrenia, as 2/3 patients show significant improvement in positive symptoms [95, 96]. In the treatment of stable schizophrenia, continuous administration of antipsychotics is vital for recovery and the prevention of relapse [97–99]. However, the efficacy of SGAs is no better than FGAs for the negative symptoms and cognitive dysfunction in stable schizophrenia [100]. A meta-analysis of 168 randomized placebo-controlled trials denied clinically significant improvements of negative symptoms with current medications [100]. With regard to cognitive dysfunction, longitudinal evidence indicated that the mild improvements in general cognition disappear after the first two months of treatment and few effective drugs have been reported to have long-term cognitive improvements [101].

### Pharmacological Treatments for Negative Symptoms

The heterogeneity of negative symptoms in pathophysiology is universally acknowledged. Negative symptoms are classified as primary or secondary [102]. Primary negative symptoms are intrinsic to the disease, while secondary negative symptoms result from known factors including positive symptoms, affective symptoms, antipsychotic-associated extrapyramidal symptoms (EPSs) and sedation, social deprivation, and substance abuse [102]. Schizophrenia with primary negative symptoms accounts for 15%–25% in patients with schizophrenia [103]. Primary and enduring negative symptoms have been defined as deficit symptoms in schizophrenia [103]. However, psychiatrists find it rather difficult to differentiate primary from secondary negative symptoms in clinical practice [104]. The term “predominant negative symptoms” is frequently used instead, as it is easy to classify according to the scale scores [105]. To our knowledge, effective pharmacological treatments for primary negative symptoms are still rare, while secondary negative symptoms seem to have more available treatments such as clozapine [106]. However, proposals for clinical management of the negative symptoms have been improved with the development of new dopamine antagonists such as cariprazine [107]. Cariprazine is proposed as a first-line treatment for patients with predominant negative symptoms, and low-dose amisulpride is an alternative when cariprazine is ineffective [107]. In general, pharmacological treatments for negative symptoms include dopamine antagonists (cariprazine and low-dose amisulpride) as monotherapy, with serotoninergic or noradrenergic and glutamatergic drugs as adjunctive therapy (Table 1).

**Table 1:** Medications for negative symptoms in schizophrenia.

Molecular target	Example compound	Clinical evidence
Dopamine antagonists	Amisulpride (low dose), monotherapy	Improvements in negative symptoms with improvements in depressive symptoms
	Cariprazine, monotherapy	Improvements in predominant negative symptoms
	lisdexamfetamine dimesylate, add-on therapy	Improvements in predominant negative symptoms
Serotoninergic and noradrenergic drugs	SSRIs , TCAs, etc. add-on therapy	Improvements of negative symptoms with improvements in depressive symptoms
Glutamate agonists	Glycine, glycine transporter-1 inhibitors, D-serine , D-amino acid oxidase (DAAO) inhibitors, D-cycloserine (DCS) add-on therapy	Small to moderate effects
Sigma-2 antagonist and 5-HT2A antagonist	Roluperidone (MIN-101) monotherapy	Positive results
Other targets	Minocycline, add-on therapy	Positive results

#### Dopamine Antagonists as Monotherapy

As noted above, deficits in dopamine transmission in mesocortical pathways are the universally accepted mechanisms for the negative symptoms. New dopamine antagonists with preferential selectivity for D3 receptors (such as low-dose amisulpride and cariprazine) have potential to relieve predominantly negative symptoms in schizophrenia.

Low-Dose Amisulpride (50–300 mg/day)

Amisulpride is a second-generation antipsychotic of the benzamide class with high affinity for dopamine D2 and D3 receptors. Amisulpride was revealed as the only antipsychotic effective in the treatment of predominant negative symptoms [108]. In the last century, low-dose amisulpride (50–300 mg/day) was highlighted for significantly reducing the scores on the Scale for the Assessment of Negative Symptoms (SANS) in the placebo-controlled studies [109–112]. Low-dose amisulpride remarkably improves predominant negative symptoms, especially avolition, attentional impairment, and retardation [112], and it is suspected to relieve non-persistent negative symptoms in medium-term treatment [109, 113]. Recently, a Chinese study reported that amisulpride (average 623.9 mg/day) reduces Positive and Negative Syndrome Scale (PANSS) negative symptom scores from baseline to week 8 with a 45.2% average reduction in Chinese patients with predominantly negative symptoms [114]. All of these studies excluded the effects of amisulpride on negative symptoms secondary to the positive symptoms and EPS by comparing the simultaneous changes in these two symptoms [113]. However, the improvement in negative symptoms is accompanied by improvements in depressive symptoms [113]. Thus, its effects on negative symptoms secondary to depression were not excluded and thus confused its effects on primary negative symptoms. Besides, its marked efficacy has only been revealed in studies by comparison with placebo [109–111], and merely a trend in favor of amisulpride has been reported when compared with other antipsychotics [115].

Cariprazine

Cariprazine is a dopamine D3 and D2 receptor partial agonist that preferentially binds to D3 receptors and a partial agonist of the serotonin 5-HT1A receptor. In 2014, the efficacy of cariprazine in the acute exacerbation of schizophrenia was explored for the first time. A meta-analysis in 2018 summarized 4 randomized controlled trials on the efficacy and safety of cariprazine in the acute exacerbation of schizophrenia [116–119]. Cariprazine seems to have equal effects on positive and negative symptoms compared with placebo (PANSS total scores, standardized mean difference (SMD) = −0.37; PANSS positive, SMD = −0.32; PANSS negative, SMD = −0.32) [120]. Cariprazine has broad-spectrum effects in acute schizophrenia, improving all 5 PANSS factor domains [121]. Although cariprazine has a higher incidence of treatment-emergent events and akathisia *versus* placebo [120], long-term administration (up to 9 mg/day) has been reported to be reported generally safe and well-tolerated by patients [122]. Only small changes in metabolic parameters and a decrease in prolactin level have been reported in cariprazine treatment compared with placebo [118, 123].

Despite its application in acute schizophrenia, cariprazine has been characterized as effective for predominant negative symptoms under the condition that the secondary negative symptoms are well-controlled [124, 125]. A rigorously-designed study published in *Lancet* compared the efficacy of cariprazine with risperidone monotherapy and found that cariprazine has advantages over risperidone in improving predominant negative symptoms as well as community functioning [125]. Cariprazine was reported to take effect at week 14 and continued to improve symptoms at the week 26 endpoint [125]. The secondary negative symptoms were well-controlled in this study, as no significant changes in positive symptoms, EPS, or depressive symptoms were detectable in the cariprazine and risperidone groups [125, 126]. However, this study had some limitations, such as the lack of a placebo control group. Another group conducted *post hoc* analyses of individual PANSS items and PANSS-derived factors. They found that cariprazine is superior to risperidone in most PANSS Negative Subscale items and across all PANSS-derived factors, which means that cariprazine has broad-spectrum efficacy for predominant negative symptoms [126].

#### Other Dopaminergic Drugs

Other preferential D3 receptor antagonists such as F17464 and ABT-925 have reached clinical study phase. Only F17464 exhibits a good efficacy in acute schizophrenia [27, 127], but without special effects on negative symptoms.

Asenapine, an agent binding to dopamine, serotonin, noradrenaline, and histamine receptors, is more effective than risperidone and haloperidol on negative symptoms [128, 129]. But it fails to outperform olanzapine in patients with persistent negative symptoms [130, 131].

Dopamine agonists (such as methylphenidate, amphetamine, and lisdexamfetamine) may improve the negative symptoms in patients with schizophrenia. A meta-analysis in 2019 on pro-dopaminergic drugs including armodafinil, L-dopa, and pramipexole found that they do not significantly improve negative symptoms [132]. However, adjunctive lisdexamfetamine dimesylate therapy improved predominant negative symptoms in outpatients with schizophrenia without positive symptom worsening [133]. Besides, the D1 dopamine agonist dihydrexidine (DAR-0100) failed to elicit significant improvements in cognitive dysfunction [134].

#### Serotoninergic and Noradrenergic Drugs (Antidepressants) as Adjunctive Therapy

Since abnormal serotoninergic or noradrenergic transmission plays an important role in negative symptoms, adjunctive antidepressants can improve these symptoms and general functioning in patients with schizophrenia. A meta-analysis in 2010 that included SSRIs, mirtazapine, reboxetine, mianserin, trazodone, and ritanserin confirmed the moderate effects of antidepressants in improving negative symptoms in chronic schizophrenia (SMD = –0.48) [135]. Another meta-analysis in 2016 found that antidepressants have small positive effects on both depressive symptoms (SMD = –0.25) and negative symptoms (SMD = –0.30) [136]. In the analysis of subgroups, the authors found that antidepressants have larger effects on predominant negative symptoms (SMD = –0.58) [136]. Antidepressants (MAOs, NDRIs, NRIs, SNRIs, SSRIs, TCAs and TeCAs, and SSRIs and TCAs) have been reported to be more efficacious on negative symptoms compared with placebo [136]. Among individual drugs, selegiline, duloxetine, citalopram, fluvoxamine, and mirtazapine have been reported to benefit the negative symptoms in schizophrenia [136]. In addition, a meta-analysis on adjunctive antidepressants in stable antipsychotic treatment reported that these drugs have small-to-medium effects on negative symptoms [137]. Of note, their beneficial effects on negative symptoms seem to be limited to the augmentation of first-generation antipsychotics [137]. The immune mechanisms underlying SSRI augmentation have been revealed with a decrease in C-reactive protein and interleukin-6 levels [138]. However, the effects of adjunctive antidepressants in schizophrenia cannot rule out the improvement of negative symptoms secondary to depression. When patients with depression are excluded, antidepressants such as fluoxetine fail to present positive results in primary and enduring negative symptoms [139].

Roluperidone (MIN-101), an antagonist of both sigma-2 and 5-HT_2A_ receptors, is a serotoninergic drug rarely used as monotherapy for schizophrenia [140]. The efficacy of both doses (32 and 64 mg) of roluperidone monotherapy on negative symptoms was confirmed in patients with stable positive symptoms [141]. The *post hoc* analysis for this trial showed that roluperidone works on the two domains, reduced experience and reduced expression, among the negative symptoms [142]. Roluperidone may also improve personal and social adjustments in patients with stable positive symptoms and concurrent clinically significant negative symptoms [143]. In these studies, the effects of positive symptoms were controlled as no significant differences were found between roluperidone and placebo [141]. However, other compounding factors in secondary negative symptoms have not yet been taken into consideration. Besides, roluperidone monotherapy improves cognitive dysfunction in stable schizophrenia as well [144].

#### Glutamatergic Drugs as Adjunctive Therapy

Abnormal glutamate signaling in the pathology of schizophrenia includes hypofunction of NMDA receptors in GABAergic interneurons and hyperglutamatergic signaling in excitatory pyramidal neurons in cortex [145, 146]. Thus, the therapeutic strategy is to augment NMDA receptor function; this includes agents targeting NMDA receptors and AMPA receptors, positive allosteric modulators of metabotropic glutamate receptors, and antioxidants [147]. Agents targeting GluN1 (a subunit of NMDA receptors) include glycine and glycine transporter-1 inhibitors, D-serine and D-amino-acid oxidase inhibitors, D-cycloserine (DCS), and KAT inhibitors [148, 149]. Adjunctive glutamatergic drugs have small to moderate effects on the negative symptoms in patients with schizophrenia. A meta-analysis including DCS and D-serine in 2013 reported a moderate effect-size improvement (SMD = 0.62). Besides, a meta-analysis that included D-serine, sarcosine, NAC, DCS, and glycine in 2011 and another that included glycine, D-serine, DCS, and sarcosine in 2010 both reported a small effect-size (2013: SMD = –0.27; 2010: SMD = 0.38) [150–152]. However, the effects of glutamatergic drugs on negative symptoms were not classified into primary or secondary negative symptoms. Although their spontaneous effects on positive symptoms are relatively small compared with negative symptoms, studies on their effects on predominant negative symptoms with well-controlled secondary negative symptoms are clearly warranted [150–152]. In terms of cognitive dysfunction, some cognitive domains such as working memory can be improved by glutamatergic drugs [153], and some glycine transporter-1 inhibitors, BI-425809, and cannabidiol are still being further explored for cognitive improvements [154].

#### Other Drugs and Dietary Supplements

Minocycline, a second-generation tetracycline, can serve as adjunctive therapy for the negative symptoms *via* reducing pro-inflammatory cytokines [155]. The early research on minocycline focused on its effects in the early phase of schizophrenia and reported some positive results [156–158]. However, a recent study denied its effects in first-episode psychosis as no progressive inflammatory process underpins the negative symptoms [159]. The administration of minocycline with risperidone demonstrated its efficacy on the negative symptoms in stable schizophrenia, which are positively co-related with the dose of minocycline [155, 160].

Sulforaphane, a natural compound extracted from the seeds and sprouts of cruciferous plants, can protect brain cells from inflammation and oxidative stress [161]. The protective mechanisms of sulforaphane are related to kelch-like ECH-associated protein 1 and the transcription factor NF-E2-related factor 2 [162, 163]. Two studies have been published on the efficacy of sulforaphane associated with schizophrenia [164, 165]. One reported that sulforaphane-rich broccoli sprout extract (30 mg/day) improves cognitive dysfunction in patients with schizophrenia [164], and the other focused on the increased brain GSH level after the administration of sulforaphane for 7 days [165].

Since reduced blood omega-3 fatty-acids are associated with cognitive dysfunction, their administration may improve this symptom cluster [166]. Besides, omega-3 fatty-acids might have better efficacy when administered at the early stages of schizophrenia [167]. Of note, they also have beneficial effects on metabolic syndrome, such as reducing the triglyceride levels in patients with schizophrenia [168]. However, more clinical studies and meta-analyses are needed, as some trials reported that they are not efficacious on cognitive dysfunction [169]. The pharmacological entities for cognitive dysfunction described above are listed in Table 2.

**Table 2:** Medications for cognitive dysfunction in schizophrenia.

Molecular target	Example compound	Clinical evidence
D1 Dopamine agonists	Dihydrexidine (DAR-0100), monotherapy	Negative proof-of-concept trial
Glutamate agonists	Glycine, glycine transporter-1 inhibitors, D-serine , D-amino acid oxidase (DAAO) inhibitors, D-cycloserine (DCS) add-on therapy	Improvement in working memory domain; mixed results.
Sigma-2 antagonist and 5-HT2A antagonist	Roluperidone (MIN-101), monotherapy	Positive results
Antioxidants	Sulforaphane, omega-3 fatty acids add-on therapy	Mixed results

### Physical Therapy for Negative Symptoms and Cognitive Dysfunction

Since cortical excitation/inhibition imbalance plays a vital role in negative symptoms and cognitive dysfunction, physical therapy may directly reverse the aberrant neural activity and cortical excitability in schizophrenia. Physical therapy for schizophrenia (especially treatment-resistant schizophrenia) includes transcranial magnetic stimulation (TMS), transcranial direct-current stimulation (tDCS), and modified electroconvulsive therapy (mECT) [170]. Compared with TMS and tDCS, mECT serves as an add-on therapy, mainly for refractory schizophrenia with little marked advance in the last decade [171, 172]. Thus, we discuss TMS and DCS below, and these two invasive forms of stimulation in the frontal cortex exhibit great potential, especially for negative symptoms [173].

#### Transcranial Magnetic Stimulation

TMS can probe the functioning of the neural networks and neurotransmitter systems associated with the negative symptoms and cognitive dysfunction in schizophrenia [174]. Deep repetitive TMS applied to the prefrontal cortex (PFC) is a common, long-accepted therapy for treatment-resistant depression [175]. Currently, this strategy is recognized as an adjunctive therapy for the negative symptoms and cognitive dysfunction in schizophrenia, especially for negative symptoms [174, 176, 177]. The first study of TMS in schizophrenia found that 20 daily H1-coil deep TMS treatments (20 Hz, 120% motor threshold) significantly reduce the negative symptoms *versus* baseline *via* assessing with the SANS [178]. However, the active and sham stimulation groups did not differ at the endpoint [178]. High-frequency (18 Hz) bilateral deep stimulation applied over the lateral PFC using the Brainsway H-2 coil has been reported to have good efficacy for negative symptoms [179]. This study excluded the effects of depression as no differences were detected in patients with or without depression [179]. Cerebellar vermal magnetic stimulation significantly improves the negative symptoms and depressive symptoms compared with sham stimulation [180]. In some studies, TMS was applied to the DLPFC to demonstrate its effects on negative symptoms. Bilateral 10-Hz stimulation of the DLPFC for 3 weeks reduced scores measured in the SANS [181]. However, repetitive TMS to the left DLPFC affects the positive and total psychotic symptoms rather than negative symptoms in patients with predominant negative symptoms [182].

In the treatment of cognitive dysfunction, improvements in working memory have been reported with high-frequency repetitive TMS over the left PFC [183]. In addition, 10-Hz rTMS stimulation of the PFC significantly improves facial affect recognition, which plays a critical role in social cognition [184]. However, some studies reported negative results. For example, 10-Hz repetitive TMS applied to the left DLPFC for 3 weeks did not improve any of the cognitive domains in schizophrenia patients with predominant negative symptoms [185]. Although some remarkable findings have been reported with TMS, a review reported insufficient evidence to support or refute the use of TMS in schizophrenia [176, 186], as its overall beneficial effects on negative and cognitive symptoms may be inconsistent and small in schizophrenia [174, 187, 188].

#### Transcranial Direct Current Stimulation

tDCS is a potential treatment for negative symptoms [189–192] and cognitive dysfunction [193, 194] in schizophrenia. Its primary effects on neurons are to shift the resting membrane potential towards depolarization or hyperpolarization [195]. In contrast to TMS, tDCS works on active neurons and cannot activate neurons in the resting state [195].

Leandro *et al*. found that patients receiving active tDCS show a marked reduction of negative symptoms *versus* controls, and the active group had higher response rates (40%) for negative symptoms *versus* the sham group (4%) (P < 0.001) [192]. Moreover, tDCS can help outpatients to improve adherence [196, 197]. The application of tDCS to the prefrontal cortex during task training may have surprisingly beneficial effects on behavioral performance [198].

In terms of cognitive dysfunction, tDCS targeting the DLPFC can improve several domains of cognition. Anodal stimulation improves attention and working memory, while cathodal stimulation improves cognitive ability and memory [194]. Of note, the working memory and overall cognition scores of patients improve over time after stimulation [193]. Besides, the mechanisms of its pro-cognitive effects have been explored *via* functional MRI [199].

In treatment-resistant schizophrenia, tDCS-clozapine combination might be an effective intervention in clozapine-refractory patients [195]. Fronto-temporal transcranial tDCS improved the awareness of illness and positive symptoms but not auditory verbal hallucinations [200]. Furthermore, add-on tDCS demonstrated efficacy on auditory hallucinations in hospitalized patients with ultra-treatment-resistant schizophrenia [190]. In all, the overall effects on both symptoms failed to elicit positive results, but the high stimulation frequency, twice daily subgroup showed improved negative symptoms (SMD = –0.31) in the latest meta-analysis of tDCS [201].

### Psychosocial Treatments

Psychosocial treatments for schizophrenia include individual psychological, psychoeducational, and family interventions [14, 29]. Among them, social skill training (SST) plays a critical role in improving the negative symptoms. SST has been demonstrated to have significant efficacy for negative symptoms compared with active controls, treatment-as-usual, and waiting list controls in two meta-analyses [202, 203]. Of note, the efficacy of SST on negative symptoms is comparable with other interventions, including medications [203]. However, more rigorously-designed studies on SST are needed to promote its application in psychological intervention [204]. In addition to SST, cognitive remediation had small to moderate beneficial effects on negative symptoms, but robust effects on cognitive dysfunction in a network meta-analysis [205, 206]. Meditation-based mind-body therapies had small to moderate efficacy on negative symptoms compared to treatment-as-usual or non-specific control interventions [207].

## Conclusions

Schizophrenia is a polygenic disease of unknown etiology. With advances in sequencing and detection technology, genetic and epigenetic analyses can promote our comprehension of the pathogenesis of schizophrenia as well as the mechanisms underlying the negative symptoms and cognitive dysfunction in schizophrenia. Currently, the treatments of negative symptoms and cognitive dysfunction play key roles in the full recovery of patients with schizophrenia. The prospects for predominant negative symptoms are good, with the advent of new D3-selective dopamine antagonists such as cariprazine. Besides, some physical therapies have the potential to improve negative symptoms and cognitive dysfunction as well. Compared with other reviews on the negative symptoms [29, 104] or cognitive dysfunction in schizophrenia [154], in this review we report new advances in physical therapy as well as genetic and epigenetic mechanisms underlying these two symptoms.

However, further exploration is still required for curing the negative symptoms and cognitive dysfunction in schizophrenia. The potential reasons for the unsatisfactory therapeutic effects of novel medications on these two symptoms might be due to their multiple domains, and the complicated mechanisms underlying each domain. Exploration of these mechanisms and treatments according to domain criteria (Research Domain Criteria, above) is anticipated. Besides, clinical studies should distinguish the heterogeneity of negative symptoms when assessing the real efficacy of novel drugs.
